# Identification of biomarkers related to γ-aminobutyric acid-associated genes in membranous nephropathy based on transcriptome data and clinical experiments

**DOI:** 10.1186/s41065-026-00681-y

**Published:** 2026-04-22

**Authors:** Jie Luo, Min Chen, Jing Li, Yue Qi, Tingyu Chen, Huibin Nie

**Affiliations:** Department of Nephrology, Chengdu Integrated TCM&Western Medicine Hospital, Chengdu, 610095 China

**Keywords:** Membranous nephropathy, Gamma-aminobutyric acid, Immune infiltration analysis, GSEA, GSVA

## Abstract

**Background:**

Membranous nephropathy (MN) is a leading cause of nephrotic syndrome in adults, being driven by incompletely understood autoimmune mechanisms. Emerging evidence suggests that γ-aminobutyric acid (GABA), traditionally considered a neurotransmitter, also functions as a critical immunomodulator in peripheral tissues. Dysregulation of GABAergic signaling has been linked to altered immune cell trafficking and metabolic stress in renal pathologies. However, its specific role and signature genes in MN pathogenesis have not been systematically characterized. This study aimed to identify novel GABA-associated biomarkers for MN and elucidate their potential mechanistic roles.

**Results:**

By integrating differential expression analysis, weighted gene coexpression network analysis, and machine learning algorithms on public transcriptomic datasets, we identified three robust GABA-associated candidate biomarkers: *AP2S1*, *STXBP1*, and *GNGT2*. These genes were consistently upregulated in patients with MN across both the training and validation cohorts. Functional enrichment analysis revealed their significant involvement in vesicle trafficking, cytochrome P450 metabolism, and cell adhesion pathways. Immune infiltration analysis demonstrated that these biomarkers had strong positive correlations with central memory CD4 + T cells and myeloid-derived suppressor cells. Furthermore, molecular docking simulations indicated high binding affinities between these targets and potential therapeutic compounds, including valproic acid and genistein. Crucially, reverse transcription quantitative polymerase chain reaction validation in an independent clinical cohort (*n* = 10) confirmed significantly elevated expression of *AP2S1*, *STXBP1*, and *GNGT2* in the peripheral blood of patients with MN versus healthy controls (*P* < 0.05).

**Conclusion:**

This study established a distinct GABAergic signature in MN, highlighting *AP2S1*, *STXBP1*, and *GNGT2* as promising noninvasive diagnostic biomarkers. Our findings suggested that GABA-related vesicle trafficking and metabolic pathways contribute to the autoimmune microenvironment in MN. These genes offer new avenues for early diagnosis and represent potential therapeutic targets for precision medicine strategies in managing MN.

**Supplementary Information:**

The online version contains supplementary material available at 10.1186/s41065-026-00681-y.

## Introduction

Membranous nephropathy (MN) is a primary glomerular disease mainly characterized by immune complex deposition under epithelial cells in the glomerular basement membrane1. MN is an important cause of nephrotic syndrome in adults, and its incidence rate has been increasing over time2. The incidence rate of MN is approximately 8–10 per million people, and the disease is more common in men than in women, especially among people older than 40 years. MN is classified as either primary (PMN) or secondary MN (Glassock 2010). Primary MN (PMN) is considered an antibody-mediated autoimmune disease. Secondary MN occurs in association with other diseases such as systemic lupus erythematosus, infections, and malignancies Biopsy [1] is the gold standard for MN diagnosis. The diagnosis of PMN relies on pathological examination. In 2009, BECK et al. identified PLA2R as a specific antigen of PMN using western blotting and mass spectrometry. In 2021, the guidelines released by Kidney Disease: Improving Global Outcomes (KDIGO) made significant revisions to the PMN diagnostic process, and renal biopsy was not long required to confirm a PMN diagnosis in patients with the clinical manifestations of nephrotic syndrome and positive serological tests for anti-PLA2R antibodies. Some potential biomarkers with important roles in the pathogenesis, diagnosis, treatment, and prognosis evaluation of MN have been reported [1]. At present, MN is mainly divided into low-, moderate-, and high-risk subtypes. Low-risk patients mainly receive conservative treatment [2, 3]. Patients with moderate- and high-risk MN are usually treated with glucocorticoids combined with cytotoxic alkylating agents or cyclosporine, rituximab monotherapy, and other drugs [4]. Even if remission is achieved, 5%–28% of patients experience relapse [5]. Despite advancements in the treatment of MN, some patients do not respond to existing treatments or experience repeat relapses, and long-term management is difficult. This highlights the need for further research to facilitate the development of more effective treatment methods.

γ-Aminobutyric acid (GABA) is the most important inhibitory neurotransmitter in the central nervous system [6]. It inhibits action potential generation by binding to postsynaptic GABA receptors, regulating ion channels, and causing cell hyperpolarization [7]. GABA plays crucial roles in regulating neuronal activity, reducing stress, relieving anxiety, and improving sleep [8]. GABA is also present in various non-neural tissues, such as the kidneys, liver, and intestines [6]. GABA exerts effects on MN through three aspects. First, GABA can regulate the immune system and alleviate inflammatory responses. In MN, immune-mediated inflammation is among the important factors leading to renal injury. Second, GABA can help alleviate glomerular inflammatory damage by inhibiting the expression of inflammatory mediators [9]. Third, GABA has antioxidant properties, which can reduce oxidative stress and help protect kidney cells from oxidative damage [10, 11]. In animal experiments, GABA has been proven to alleviate nephrotoxicity caused by the chemotherapeutic agent cisplatin, highlighting its protective effects on the kidneys [9]. Collectively, these lines of evidence suggest that dysregulation of GABAergic signaling might contribute to the pathogenesis of MN, making GABA-related genes compelling candidates for biomarker discovery [12, 13].

Therefore, this study analyzed differentially expressed genes (DEGs) between patients with MN and normal controls using transcriptomic data to discover potential biomarkers related to GABA in MN. Subsequently, a diagnostic model based on these markers was constructed, and the accuracy and utility of the potential biomarkers were experimentally verified to provide new ideas and technical support for the early detection and precise treatment of MN.

## Materials and methods

### Source of data

Data pertaining to MN were obtained from the GEO database (https://www.ncbi.nlm.nih.gov/geo/). Notably, the MN training dataset (GEO ID: GSE200828, GPL199883 platform) contained 51 glomerular tissue samples from patients with MN (disease group) and 6 glomerular tissue samples from healthy controls (control group). These data served as the training dataset. In a similar manner, the MN validation dataset (GEO ID: GSE115857, GPL14951 platform) was also retrieved from the GEO repository. It included 11 renal tissue samples from individuals with MN (affected group) and 7 renal tissue samples from healthy individuals (comparison group). In total, 97 GABA-related genes (GABAGs) were extracted from the Molecular Signatures Database (MSigDB), specifically utilizing gene sets associated with “GABAergic synapse” (Kyoto Encyclopedia of Genes and Genomes [KEGG]: hsa04727) and Gene Ontology (GO) terms related to GABA signaling (Table S1). It must be clarified that this definition relies on canonical pathway annotations primarily derived from neuronal and synaptic contexts. Consequently, in this study, the term “GABA-related” denotes a bioinformatic association based on pathway membership rather than experimentally verified functional roles within the renal microenvironment. This distinction acknowledges that although these genes are integral to GABA signaling in the nervous system, their specific involvement in kidney-specific GABAergic mechanisms remains to be verified.

### Differential expression gene analysis in GSE200828

To investigate the gene expression differences between the disease and control groups in the MN training dataset, this study employed the limma package [14] (v 3.52.4). To identify DEGs by comparing the disease group to the control group(disease group versus control group; *P* < 0.05 and |log2FC| > 0.5). To present these DEGs more intuitively, a volcano plot illustrating gene expression disparities was created using the ggpubr package (v 0.6.0, CRAN R-project).

### Identification of membranous nephropathy-associated module genes via weighted gene coexpression network analysis (WGCNA)

To identify module genes strongly associated with MN, WGCNA was executed utilizing the WGCNA package (v 1.72-5) [15] on all samples derived from GSE200828. First, the good samples genes function was used to remove low-quality gene data. The pheatmap package (v 1.0.12, https://CRAN.R-project.org/package=pheatmap) was then utilized to generate a clustering heatmap of nonoutlier samples. Subsequently, the soft threshold routine was utilized to select the optimal soft threshold (β = 1–20, R^2^ = 0.9, with a minimum R^2^ of 0.85) according to the topological model and WGCNA mean connectivity. This step enabled the construction of a scale-free network and the partitioning of module genes. Finally, Pearson’s correlation analysis was executed to compute the correlation coefficient (r) and P-values between modules and MN traits (|r| > 0.3 and *P* < 0.05). The modules exhibiting the most significant positive and negative correlations with MN were selected as key modules, and the genes within these key modules were defined as WGCNA-MN key genes (WGCNA-MNKGs).

### Identification and role evaluation of candidate genes

To identify genes associated with GABA in MN, the gvennn package (v 0.1.9) was utilized to identify the overlap of DEGs, GABAGs, and WGCNA-MNKGs to pinpoint candidate genes. Subsequently, to investigate the biological functions of these candidate genes, gene ID conv (from symbol to Entrez ID) was conducted using the org.Hs.eg.db package (v 3.18.0). Moreover, the clusterProfiler package (v 4.7.1.003) was employed to conduct GO analysis via the enrichGO function, encompassing three classifications: biological processes (BPs), molecular functions (MFs), and cellular components (CCs). Concurrently, the enrichKEGG function was applied to KEGG pathway analysis (P.adjust < 0.05). Ultimately, the GOplot package (v 1.02) was used to generate chord diagrams to visualize the outcomes.

In addition, to deeply investigate the interplay among proteins specified by the candidate genes, the potential genes were incorporated into the STRING database (https://string-db.org/), and the species was set as *Homo sapiens* to construct a protein–protein interaction (PPI) network (confidence ≥ 0.4).

### Identification of candidate biomarkers

To pinpoint and secure potential biomarkers, 5-fold cross-validated least absolute shrinkage and selection operator (LASSO) regression analysis was initially conducted on the potential genes within the MN training dataset using the glmnet package (version 4.1-4) [16]. Subsequently, support vector machine recursive feature elimination (SVM-RFE) analysis was conducted on the candidate genes using the e1071 package (v 1.7–14, https://CRAN.R-project.org/package=e1071), yielding a set of genes selected by SVM-RFE. Finally, the ggvenn package (v 4.3.0) was used to identify the intersection of the LASSO-selected genes and SVM-RFE–selected genes, and the genes in the intersection were designated as candidate biomarkers.

### Identification of biomarkers

To pinpoint biomarkers, one-tailed Wilcoxon rank-sum tests were employed to evaluate the expression of potential biomarkers across disease and control groups within both the MN training and validation datasets. Subsequently, the outcomes were graphically represented using the ggplot2 package (v 3.5.1) [17]. Ultimately, the potential biomarkers exhibiting consistent expression patterns and significant expression differences between groups (*P* < 0.05) in both datasets were chosen as biomarkers.

### Development and confirmation of a diagnostic biomarker model

To evaluate the predictive power of biomarkers for MN development, the rms package (v 6.5-0) was implemented to generate a scoring chart in GSE200828. Each biomarker’s score was aggregated to determine the overall score, with a higher score signifying an increased likelihood of MN. Then, the regplot package (v 1.1) was applied to illustrate a calibration curve that assessed the forecasting precision and clinical validity of the biomarkers using a significance threshold of *P* > 0.05. Concurrently, the rmda package (v 1.6) was utilized to depict a decision curve to assess the clinical value of the scoring chart model.

### Gene set enrichment analysis and gene set variation analysis

To explore the underlying biological mechanisms and pathways associated with biomarkers in MN, the psych package (v 2.2.9) was utilized to conduct Spearman’s correlation analysis among the biomarkers and the remaining genes within GSE200828. The genes were subsequently ordered according to the criteria of |ρ| ≥ 0.3 and *P* < 0.05. Subsequently, the clusterProfiler package (v 4.7.1.003) was employed for GSEA. c2.cp.kegg.v2022.1.Hs.symbols.gmt was sourced from MSigDB as the reference gene set. Pathways were filtered according to the criteria of false discovery rate ≤ 0.25, *P* < 0.05, and normalized enrichment score > 1. The top five enriched pathways, as determined by P-values, were selected for illustration.

To further explore the distinctions in pathways among the biomarkers, GSVA was applied to each sample within GSE200828 to pinpoint pathways that exhibited substantial variations between the disease and control groups. Initially, the gene collection c2.kegg.v7.4.symbols from MSigDB was selected as a benchmark gene collection. Thereafter, the single-sample gene set enrichment analysis (ssGSEA) function within the GSVA package (v 1.46.0) was used to determine the ssGSEA values for all gene sets across each sample. Subsequently, the limma package (v 3.52.4) was utilized to assess the variations in gene set scores between the disease and control groups (*P* < 0.05, |t| ≥ 2). Ultimately, the outcomes were graphically presented using the ggplot2 package (v 3.5.1).

### Chromosome localization analysis

To clarify the location of biomarkers on chromosomes, the RCircos package (v 1.2.2) [18] was used to illustrate the expression of biomarkers on different chromosomes.

### Molecular regulation network

To elucidate the regulatory mechanisms influencing biomarkers, several databases were utilized to identify the microRNAs (miRNAs) linked to these biomarkers. The biomarkers were input into the DIANA-microT (https://dianalab.e-ce.uth.gr/microt_webserver/), miRDB database (https://mirdb.org/), miRabel (http://bioinfo.univ-rouen.fr/mirabel/), miRWalk database (http://mirwalk.umm.uni-heidelberg.de/), and TargetScan databases (https://www.targetscan.org/vert_80/). Each database generated corresponding miRNA prediction results for each biomarker based on its unique algorithm. These results were deduplicated, and the intersection of the deduplicated results was used to obtain a relatively accurate set of miRNAs corresponding to each biomarker. Subsequently, the miRNAs corresponding to biomarkers were input into the StarBase database (v 3.0, http://starbase.sysu.edu.cn/index.php) to predict long noncoding RNAs (lncRNAs), and the prediction results were also deduplicated. Finally, the SangerBox tool (http://sangerbox.com/) was used to construct an mRNA–miRNA–lncRNA regulatory network diagram.

### Analysis of immune cell infiltration

To assess the distribution of immune cells in the disease and control groups within GSE200828, ssGSEA was implemented to determine the infiltration levels of 28 unique immune cell types. Subsequently, the Wilcoxon rank-sum test (with a significance level of *P* < 0.05) was employed to examine the disparities in immune cell infiltration between the two groups, effectively identifying immune cells with notable differences. Moreover, to investigate the relationships between biomarkers and various immune cells, the psych package (v 2.2.9) was applied to compute the correlation coefficient (|r| > 0.3, *P* < 0.05). Lastly, the ggplot2 package (v 3.5.1) was utilized to graphically depict the aforementioned findings.

### Drug prediction and molecular docking analysis

To conduct drug prediction and molecular docking analysis, the Comparative Toxicogenomics Database (CTD, https://ctdbase.org/) was utilized to identify drugs related to biomarkers. Cytoscape software (v 3.9.1) [19] was employed to construct a targeted network diagram that included biomarkers and drugs. Subsequently, to assess the binding ability of biomarkers to small-molecule compounds, the amino acid sequences of biomarker transcription factors were obtained from UniProt (https://www.uniprot.org/), and the protein structures of biomarker transcription factors were predicted using AlphaFold (https://alphafold.ebi.ac.uk/). Meanwhile, the 2D structures of relevant drugs were downloaded from the small-molecule compound database PubChem (https://pubchem.ncbi.nlm.nih.gov/) as ligands. AutoDock software (v 4.2.6, https://autodock-vina.readthedocs.io/en/latest/index.html) and MGLTools (v 1.5.7, https://ccsb.scripps.edu/mgltools/) were used to perform molecular docking simulations and analyze the interaction forces. Finally, PyMOL (v 4.6.0, https://www.pymol.org/) was used to display the conformation after docking (score ≤ − 5 kcal/mol).

### RNA extraction and reverse transcription quantitative polymerase chain reaction (RT-qPCR)

This study was conducted in compliance with the Declaration of Helsinki, and ethical approval was obtained from the Ethical Review Board of the Chengdu Integrated TCM & Western Medicine Hospital (Approval No.: 2025YNYJ044, date of approval: July 31, 2025). The study included five patients with MN and five healthy controls, as detailed in Table 1. Patients with MN were diagnosed on the basis of clinical manifestations of nephrotic syndrome and positive serological tests for anti-PLA2R antibodies, per the 2021 KDIGO guidelines. The inclusion criteria were as follows: age ≥ 18 years, confirmed MN diagnosis, and no prior immunosuppressive therapy. Conversely, patients diagnosed with secondary MN (systemic lupus erythematosus, infections, malignancies), acute kidney injury, or concurrent kidney diseases were excluded. Patients in the control group had normal renal function (estimated glomerular filtration rate [eGFR] > 90 mL/min/1.73 m^2^), no kidney disease history, and no use of renal-impacting medications. All participants provided written informed consent prior to enrollment.

Total RNA was isolated from the blood samples of the enrolled participants using the TRIzol^®^ method. Briefly, 600 µL of blood were mixed with 700 µL of TRIzol reagent and incubated on ice for 10 min, followed by chloroform addition and centrifugation. The RNA concentration was measured using NanoDrop 50. Reverse transcription was performed using the HiFiScript III cDNA synthesis kit (Yeasen Biotech) with custom primers (Sangon Biotech). Gene expression was quantified using the 2^−ΔΔCt^ method, and GraphPad Prism 10 was employed for statistical analysis (*P* < 0.05; Table S2).

**Table 1:** Characteristics of the study participants

Partici pants	Age (years)	Sex	24-h urinary protein (g/24 h)	Plasma albumin (g/L)	eGFR (mL/min/1.73 m^2^)	Immunosuppressive agents used
Patient 1	51	Male	5.32	19.0	71.25	No
Patient 2	58	Male	3.19	30.0	56.17	No
Patient 3	55	Male	3.69	26.9	40.67	No
Patient 4	61	Male	9.96	18.9	72.88	No
Patient 5	61	Female	5.78	17.6	18.36	No
Normal 1	43	Female	—	38.0	103.81	No
Normal 2	37	Female	—	40.3	101.62	No
Normal 3	28	Male	—	41.2	123.54	No
Normal 4	22	Male	—	39.6	125.35	No
Normal 5	53	Female	—	38.5	102.76	No

## Results

### Integrated analysis uncovers 22 candidate genes for MN

**Fig. 1:**
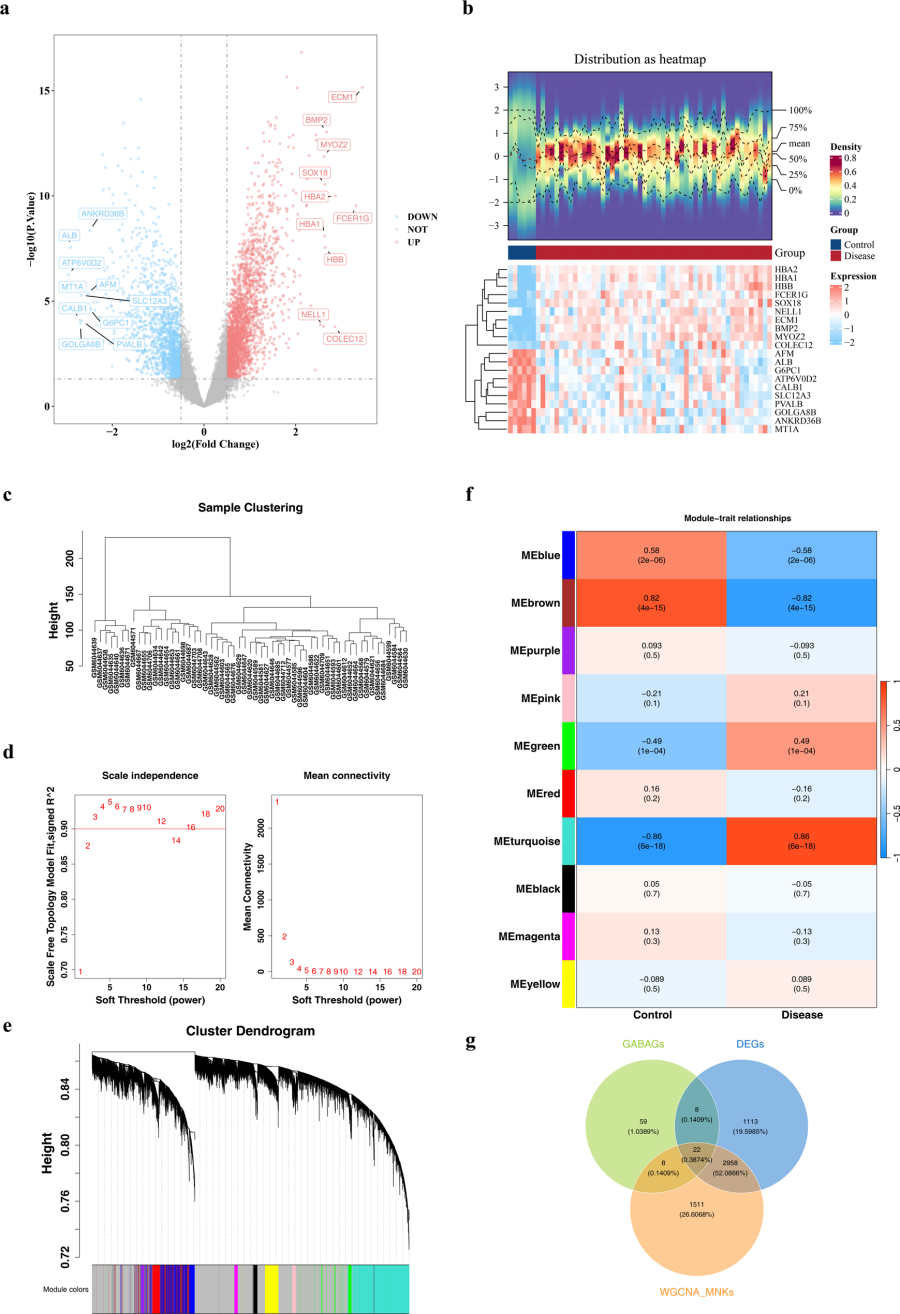
**I**ntegrated bioinformatics analysis identifies candidate genes for membranous nephropathy. **a** Volcano plot showing differentially expressed genes (DEGs) between MN patients and healthy controls. Red and blue dots represent significantly upregulated and downregulated genes, respectively (*P* < 0.05, |log2FC| > 0.5). **b** Hierarchical clustering heatmap of DEG expression profiles across all samples. The density plot above shows the distribution of gene expression levels. **c** Sample clustering dendrogram after outlier removal. **d** Selection of the soft-thresholding power for WGCNA. The left panel shows the scale-free topology fit index, and the right panel shows the mean connectivity. A power of 3 was chosen as it achieved a scale-free topology (R² > 0.9). **e** Clustering dendrogram of co-expressed gene modules identified by WGCNA. **f** Correlation heatmap between module eigengenes and MN traits. The turquoise module showed the strongest positive correlation (*r* = 0.86), while the brown module showed the strongest negative correlation (*r*=-0.82) with MN. **g** Venn diagram illustrating the overlap of 22 candidate genes derived from the intersection of DEGs, GABA-related genes (GABAGs), and WGCNA key module genes (WGCNA-MNKGs)

Differential expression analysis revealed 4101 DEGs between the disease and control groups within GSE200828. Of these, 2580 genes were upregulated, and 1521 genes were downregulated. DEGs were visualized using a volcano plot, which effectively depicted the distribution of DEGs across samples from both the disease and control groups. Significantly upregulated genes included *ECM1* and *BMP2*, and significantly downregulated genes included *ALB* and *AFM*. The majority of DEGs were predominantly located on both extremities of the plot, suggesting substantial expression disparities between the two groups (Fig. 1a).

The heatmap illustrated the gene expression patterns across various samples, with the density plot displaying the frequency distribution of gene expression. The dendrogram on the left organized genes into clusters based on their expression profiles, assembling genes with comparable expression traits into the same clusters (Fig. 1b). In WGCNA, the nonoutlier sample clustering diagram was used to present the sample clustering relationships. Low-quality data and outlier samples were removed, providing a basis for subsequent analysis (Fig. 1c). The fitting index for scale-free property and average connectedness were used to assist in determining the appropriate soft threshold. When the soft threshold was 3, R^2^ reached 0.90, meeting the conditions for constructing a scale-free network. At this time, the network had scale-free properties, and the node connections followed a power-law distribution, meaning that a few highly connected nodes played key roles. Based on this soft threshold, gene modules could be accurately divided (Fig. 1d). The module clustering dendrogram was employed to group genes based on their expression profiles, categorizing them into various modules (Fig. 1e). The module–trait correlation heatmap results revealed that the brown module had the strongest negative correlation with MN, marked by a correlation coefficient of − 0.82 and P-value of 4.07e-15. Conversely, the turquoise module exhibited the most significant positive correlation with MN, with a correlation coefficient of 0.86 and P-value of 5.73e − 18. These two modules were identified as pivotal. The 4499 genes within these critical modules were identified as WGCNA-MNKGs, which might significantly influence the development of MN (Fig. 1f). The intersections of 97 GABAGs, 4499 WGCNA-MNKGs, and 4101 DEGs were analyzed, yielding 22 overlapping genes (Fig. 1g, Table S3). These overlapping genes were considered candidate genes.

### Deciphering candidate genes: GO, KEGG, and PPI analyses

**Fig. 2:**
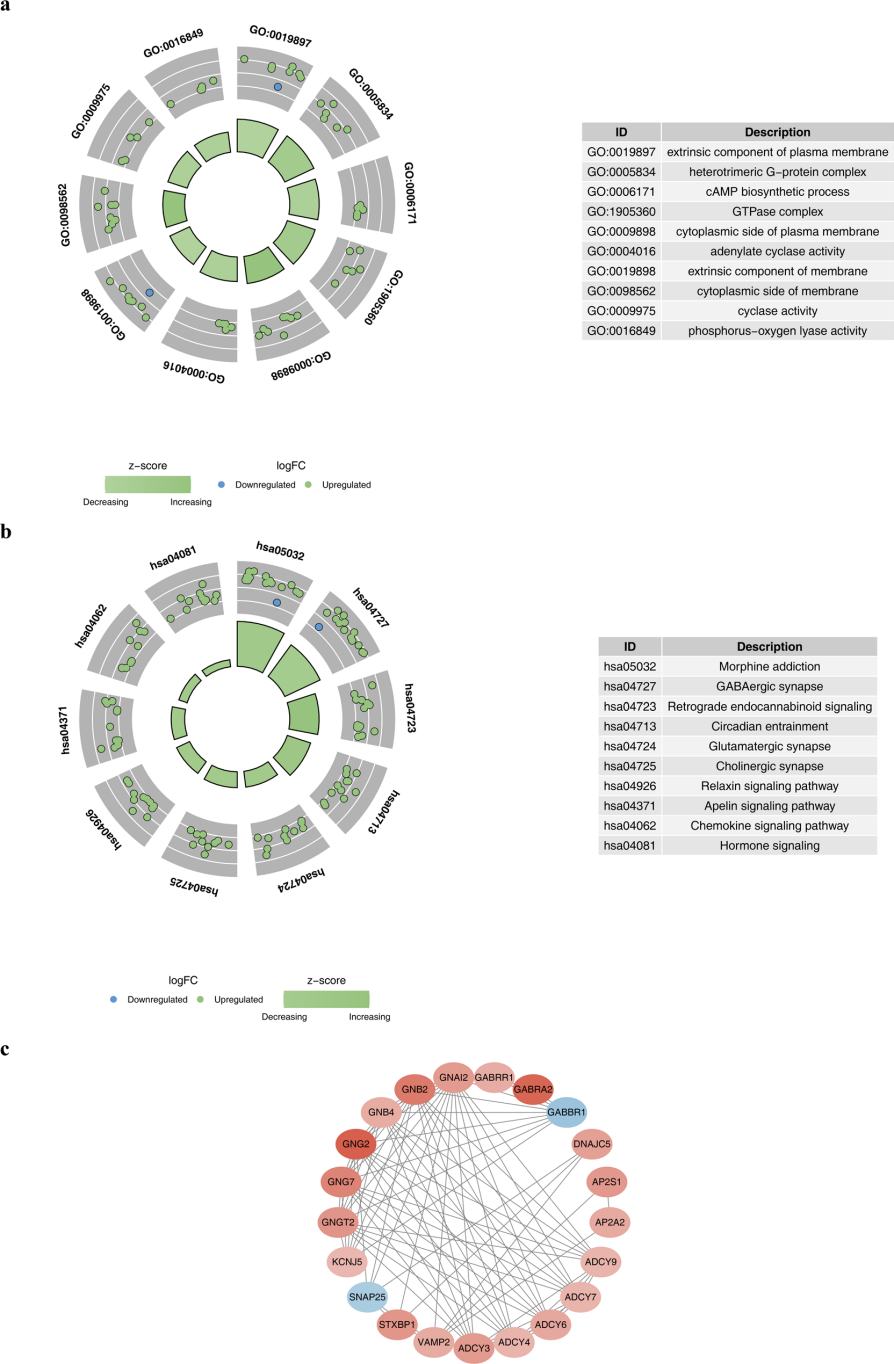
Functional enrichment and protein-protein interaction (PPI). **a** Bubble plot of Gene Ontology (GO) enrichment results for the 22 candidate genes, categorized into Biological Process (BP), Cellular Component (CC), and Molecular Function (MF). Dot size represents gene count, and color indicates significance (FDR). Key terms include “gamma-aminobutyric acid signaling pathway”. **b** Kyoto Encyclopedia of Genes and Genomes (KEGG) pathway enrichment analysis highlighting significant pathways associated with MN. The x-axis represents the Rich Factor. **c** PPI network constructed using the STRING database. Nodes represent proteins, and edges represent interactions. Hub genes with high degree scores are highlighted in larger nodes

GO enrichment analysis was conducted on the candidate genes, yielding 351 outcomes, including 209 BPs, 85 CCs, and 57 MFs. Based on the adjusted P-value, the top 10 pathways were highlighted for discussion: extrinsic component of the plasma membrane, heterotrimeric g-protein complex, biosynthetic process, GTPase complex, cytosolic aspect of the plasma membrane, adenyl cyclase activity, extrinsic part of the membrane, cytosolic aspect of the membrane, cyclase activity, and phosphorus–oxygen lyase activity (Fig. 2a). These pathways suggest that the candidate genes are predominantly engaged in the structural composition of the cell membrane, signal transduction, and critical enzymatic reactions, which are of considerable importance for cell communication, metabolic regulation, and function preservation, thus illustrating the need for future investigations into their biological roles. Additionally, KEGG functional enrichment analysis was performed on the candidate genes, identifying 78 associated pathways. The top 10 pathways were presented, namely GABAergic synapse, morphine addiction, retrograde endocannabinoid signaling, circadian rhythm, glutamatergic synapse, cholinergic synapse, relaxin signaling pathway, apelin signaling pathway, chemokine signaling pathway, and hormone signaling (Fig. 2b, Table S4). This enrichment reflects the established roles of genes in neuronal pathways as annotated in public databases, and their specific functional contributions to GABA signaling in podocytes or other renal cells have not yet been experimentally confirmed. These insights offer crucial clues for understanding the biological functions of the candidate genes.

By analyzing the PPI network constructed from the candidate genes, after removing the isolated proteins, the network contained 21 nodes with interaction relationships, and the nodes had many as 86 interaction connections, indicating the existence of a relatively complex interaction network among these proteins. Among them, *GNAI2* and *GNG2* exhibited upregulated states, whereas *GABBR1* and *SNAP25* displayed downregulated states (Fig. 2c). These upregulated or downregulated genes might participate in specific biological pathways by regulating the functions of corresponding proteins.

### Discovery of *AP2S1*, *STXBP1*, and *GNGT2* as potential biomarkers

**Fig. 3:**
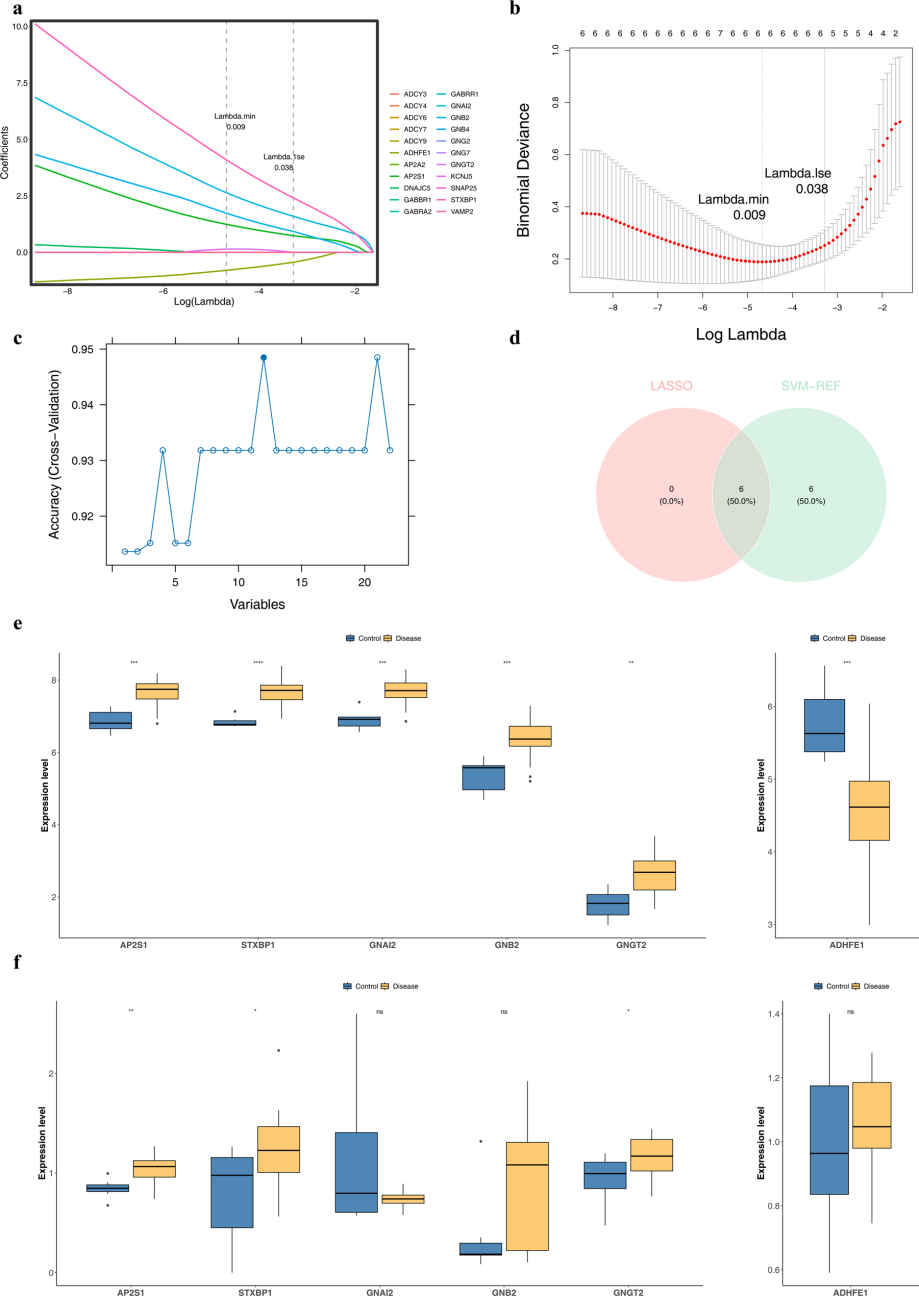
Machine learning-based screening and validation of diagnostic biomarkers. **a** LASSO coefficient profiles of the 22 candidate genes. **b** Cross-validation curve for LASSO regression showing the optimal lambda (λ) value selected via 10-fold cross-validation. **c** Accuracy curve of the Support Vector Machine-Recursive Feature Elimination (SVM-RFE) algorithm versus the number of features. **d** Venn diagram showing the intersection of genes selected by LASSO and SVM-RFE, identifying three core biomarkers: *AP2S1*,* STXBP1*, and *GNGT2.* **e** Expression levels of the three core biomarkers in the training dataset (GSE200828). *AP2S1* and *STXBP1* were significantly upregulated in MN samples. **f** Validation of core biomarker expression in the independent validation dataset (GSE115857), confirming consistent differential expression patterns. Data are presented as mean ± SD. *P* < 0.05, **P* < 0.01, **P < 0.001

First, LASSO regression analysis was conducted on 22 candidate genes. When log (lambda) reached the minimum error of 0.009, model performance was considered optimal, and six LASSO-selected genes were selected: *AP2S1*, *ADHFE1*, *STXBP1*, *GNAI2*, *GNB2*, and *GNGT2* (Fig. 3a–b). The SVM-RFE results demonstrated that the model’s prediction accuracy was highest when 12 genes were included, and thus, 12 SVM-RFE–selected genes were screened: *STXBP1*, *GNAI2*, *AP2A2*, *AP2S1*, *DNAJC5*, *GNB2*, *GABBR1*, *VAMP2*, *ADHFE1*, *GNG2*, *GABRR1*, and *GNGT2* (Fig. 3c). Subsequently, the intersection of the LASSO-selected genes and the SVM-RFE–selected genes was determined, resulting in six candidate potential biomarkers: *AP2S1*, *ADHFE1*, *STXBP1*, *GNAI2*, *GNB2*, and *GNGT2* (Fig. 3d). Finally, the transcriptional levels of these six candidate potential biomarkers were compared between the GSE200828 and GSE115857 datasets. The results indicated that the expression of *AP2S1*, *STXBP1*, and *GNGT2* was consistently higher in the disease group than in the control group in both datasets (*P* < 0.05; Fig. 3e–f). Therefore, these three genes were designated as potential biomarkers.

### Risk assessment of MN using the potential biomarker-based nomogram

**Fig. 4:**
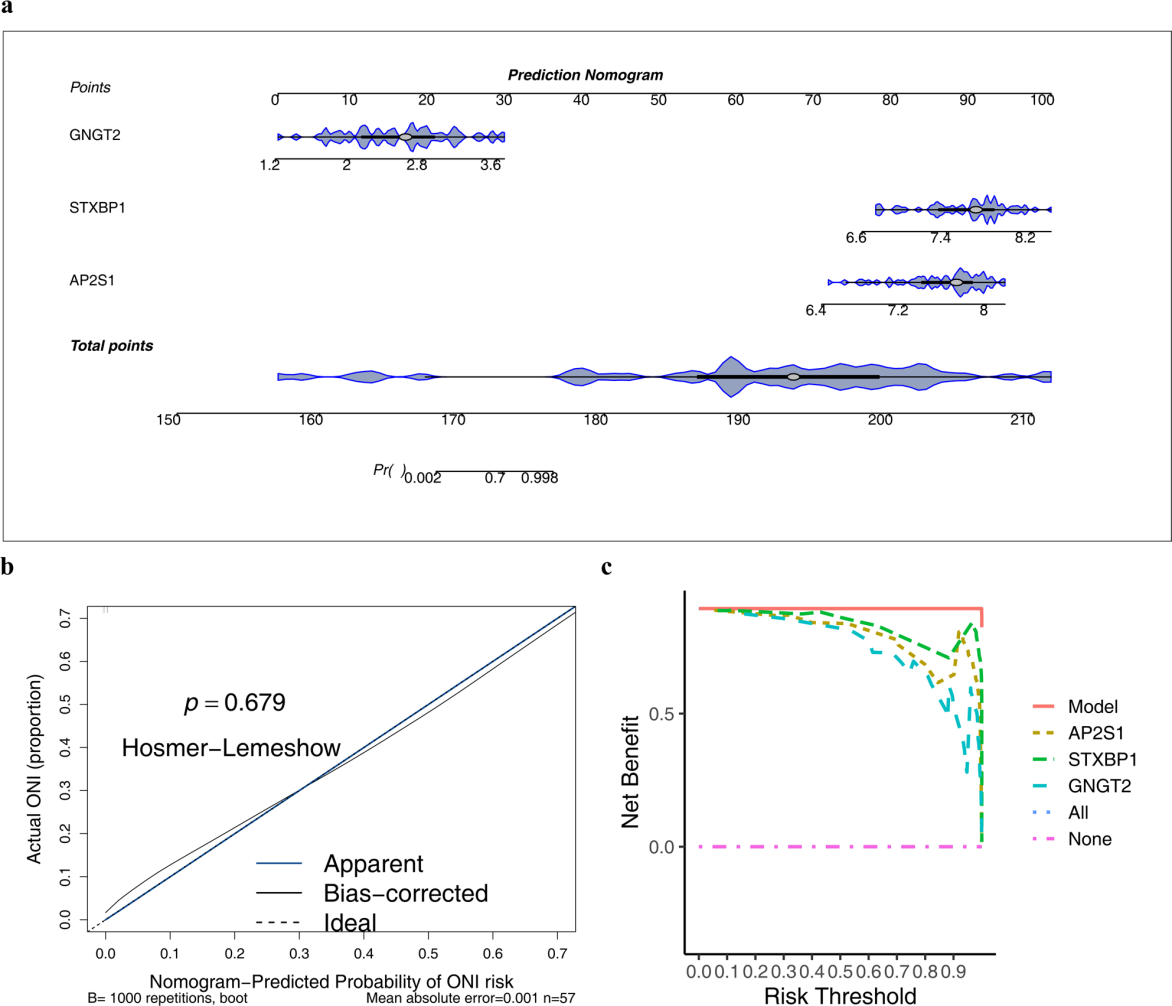
Development and evaluation of a diagnostic nomogram model based on core biomarkers. **a** Nomogram integrating the expression levels of *AP2S1*,* STXBP1*, and *GNGT2* to predict the probability of MN. Each variable is assigned a score, and the total score corresponds to the predicted risk. **b** Calibration curve assessing the agreement between the predicted probabilities and the actual observed outcomes. The diagonal dashed line represents perfect prediction. **c** Decision Curve Analysis (DCA) evaluating the clinical net benefit of the nomogram model across a range of threshold probabilities compared to “treat all” or “treat none” strategies

We utilized a nomogram to gauge the risk of MN leveraging the utility of the potential biomarkers *AP2S1*, *STXBP1*, and *GNGT2*. An individual was considered to have a relatively elevated risk of MN when the total score ranged 150–210 (Fig. 4a). We constructed a calibration curve to examine the correlation between the model’s predicted and actual probabilities. The Hosmer–Lemeshow test yielded a P-value of 0.679, and the calibration curve’s slope approximated 1, signifying substantial alignment between the predicted and actual probabilities of the model (Fig. 4b). The decision curve illustrated favorable net benefits across various risk thresholds, evidencing the model’s sound clinical predictive efficacy and practical utility (Fig. 4c).

### Pathway landscape of MN potential biomarkers: Findings from GSEA and GSVA

**Fig. 5:**
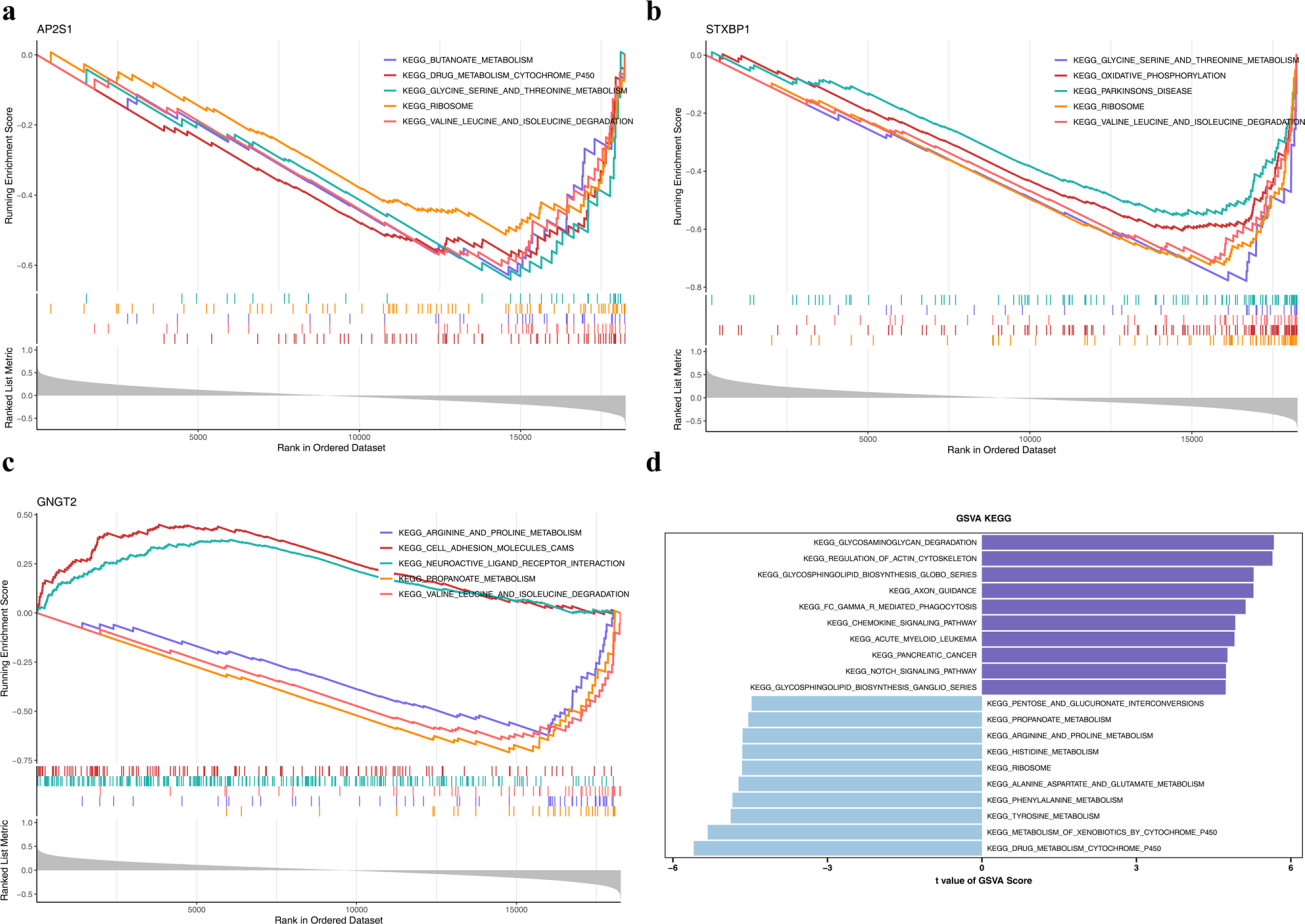
Gene set enrichment analysis (GSEA) (**a**–**c**) GSEA enrichment plots for *AP2S1* (**a**), *STXBP1* (**b**), and *GNGT2* (**c**). The plots show the enrichment of GABAergic synapse-related gene sets in samples with high expression of these biomarkers. Normalized Enrichment Score (NES) and False Discovery Rate (FDR) are indicated. **d** GSVA heatmap displaying the activity scores of top enriched pathways across MN and control samples. Red indicates high pathway activity, and blue indicates low activity. Significant differences in synaptic transmission pathways were observed between groups

GSEA indicated that *AP2S1* was prominently enriched in 65 pathways. These pathways involved the metabolism of drugs via cytochrome P450 (CYP450); the degradation of valine, leucine, and isoleucine; ribosome-associated processes; butanoate metabolism; and the metabolism of glycine, serine, and threonine (Fig. 5a). *STXBP1* was significantly enriched in 112 pathways, including the metabolism of glycine, serine, and threonine; oxidative phosphorylation; Parkinson’s disease-related processes; ribosome-related functions; and the degradation of valine, leucine, and isoleucine (Fig. 5b). *GNGT2* was notably enriched in 79 pathways, including the metabolism of arginine and proline; cell adhesion molecule-related mechanisms; the interaction between neuroactive ligands and their receptors; propanoate metabolism; and the degradation of valine, leucine, and isoleucine (Fig. 5c). These findings suggested that *AP2S1*, *STXBP1*, and *GNGT2* could potentially serve as multitarget intervention candidates by extensively participating in maintaining cellular metabolic homeostasis, regulating neural function, and participating in the pathological processes of diseases. GSVA demonstrated significant disparities in signaling pathways between the disease and control cohorts. In total, 120 pathways exhibited notable differences, including 74 upregulated and 46 downregulated pathways. The upregulated pathways included glycosaminoglycan degradation and actin cytoskeleton regulation, whereas the downregulated pathways included CYP450-mediated drug metabolism and CYP450-related xenobiotic metabolism (Fig. 5d, Table S5). These differences in signaling pathway activity imply that patients with MN might have imbalances in maintaining cellular structure and dysfunction in metabolic detoxification, laying the foundation for exploring the key regulatory nodes of the disease and potential intervention targets.

### Chromosomal positions and regulatory networks of the potential biomarkers *AP2S1*, *STXBP1*, and *GNGT2*

**Fig. 6:**
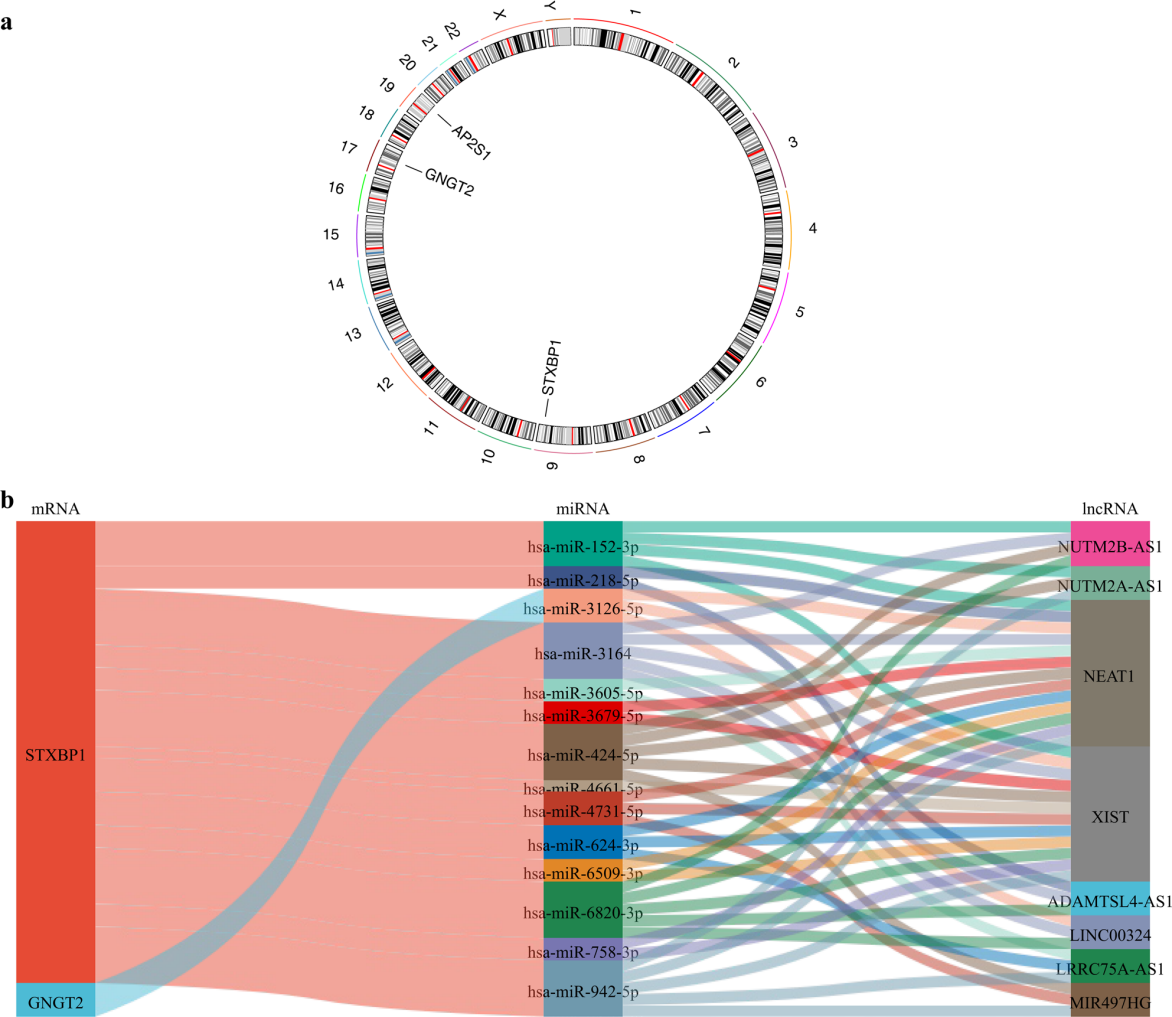
Chromosomal localization and construction of the lncRNA-miRNA-mRNA ceRNA regulatory network. **a** Chromosomal distribution of the three core biomarkers (*AP2S1*,* STXBP1*,* GNGT2*) across human chromosomes. **b** Competing endogenous RNA (ceRNA) network involving the core mRNAs, predicted miRNAs, and regulatory lncRNAs. Circles represent mRNAs, diamonds represent miRNAs, and triangles represent lncRNAs. Edges indicate regulatory interactions predicted by TargetScan and miRDB. The network suggests that specific lncRNAs may sponge miRNAs to modulate the expression of the core biomarkers in MN

Chromosomal localization analysis indicated that the potential biomarkers *AP2S1*, *STXBP1*, and *GNGT2* were located on chromosomes 19, 9, and 17, respectively (Fig. 6a). The precise chromosomal positioning of these genes is essential for understanding their genetic characteristics and roles in disease.

In this study, databases such as DIANA-micro T, miRDB, miRabel, miRWalk, and TargetScan were used to predict the miRNAs corresponding to each biomarker. After deduplicating and intersecting the genes, *AP2S1* was associated with one miRNA, *STXBP1* was associated with 48 miRNAs, and *GNGT2* was associated with six miRNAs. Subsequently, lncRNAs corresponding to the predicted miRNAs were predicted, and after removing duplicates, *STXBP1* was linked to 148 lncRNAs, and *GNGT2* was linked to six lncRNAs (Table S6). Finally, an mRNA–miRNA–lncRNA regulatory network was constructed to visualize the ceRNA interactions. Because of the strict inclusion criteria requiring miRNAs to be present across all five datasets, only *STXBP1* and *GNGT2* formed complete regulatory axes with the identified lncRNAs. Although *AP2S1* was predicted to associate with one candidate miRNA, no corresponding lncRNAs meeting our frequency threshold were identified for this specific miRNA; consequently, a complete ceRNA axis for *AP2S1* could not be established, and it was excluded from the final network visualization. The resulting network for *STXBP1* and *GNGT2* revealed complex regulatory associations (Fig. 6b), highlighting the intricate posttranscriptional control mechanisms in MN.

### Immune cell infiltration and biomarker correlation analysis in MN

**Fig. 7:**
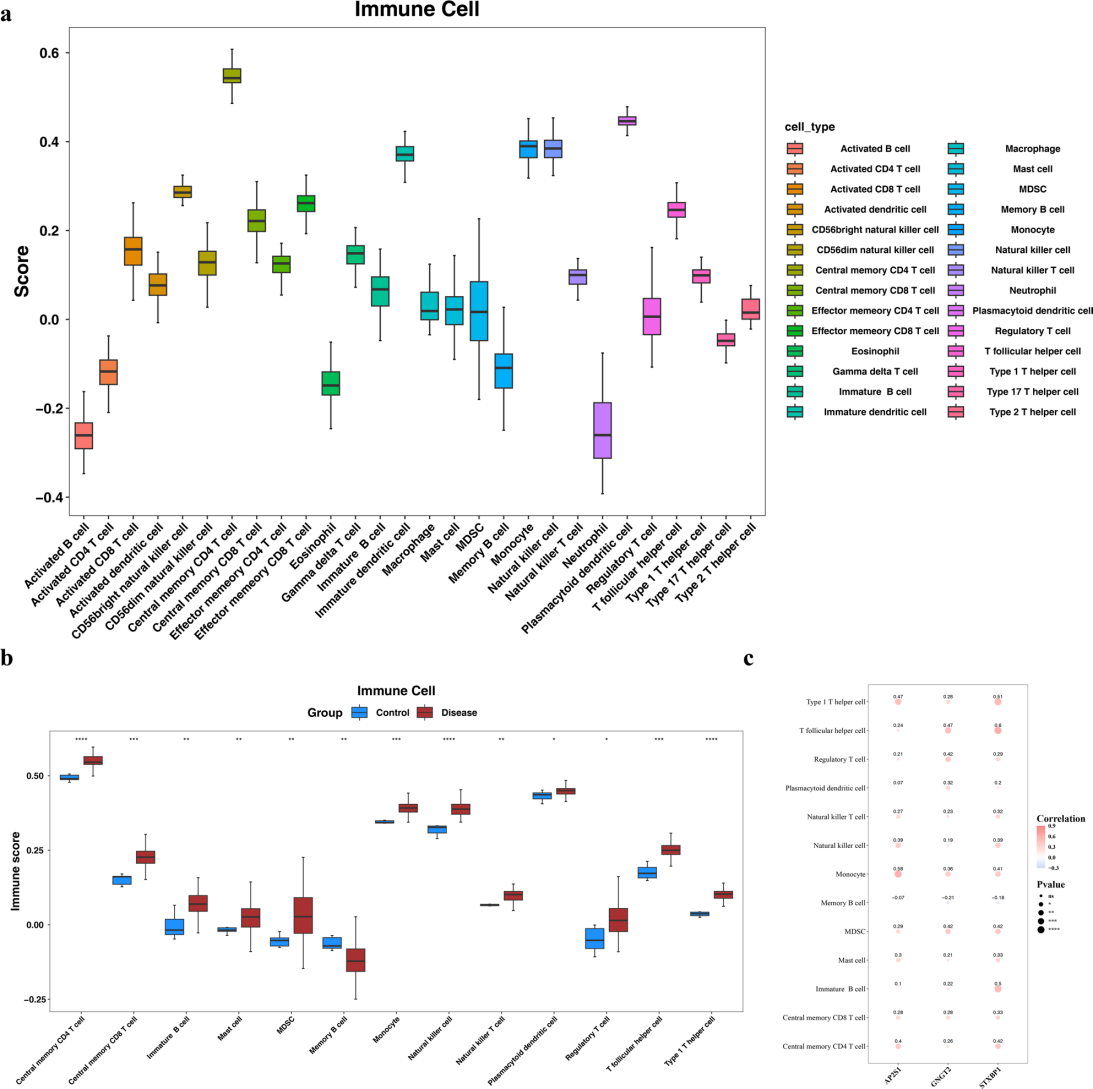
Immune cell infiltration landscape and correlation with core biomarkers in MN. **a** Stacked bar plot showing the relative composition of 28 immune cell types in all samples, estimated by the CIBERSORT algorithm. **b** Boxplots comparing immune cell scores between MN patients (red) and healthy controls (blue). Significant differences were observed in CD4 + memory resting T cells and M0 macrophages (*P* < 0.05). **c** Heatmap of Spearman correlations between the expression of core biomarkers (AP2S1, STXBP1, GNGT2) and the abundance of differentially infiltrated immune cells. Red and blue represent positive and negative correlations, respectively. *P* < 0.05, **P* < 0.01

Initially, the findings from immune infiltration analysis suggested that central memory CD4 T cells had a comparatively elevated score (median, 0.5), whereas activated B cells displayed a significantly reduced score (median, approximately − 0.3) This marked difference strongly suggests an immune imbalance among patients with MN, potentially because of irregular activation or dysfunction of central memory CD4 T cells and activated B cells, which might substantially influence the onset and advancement of MN (Fig. 7a).

Additionally, greater memory B cell infiltration was noted in the control group, whereas the other 12 immune cell types, including central memory CD4 T cells and immature B cells (*P* < 0.0001 and *P* < 0.01, respectively), were more prevalent in the disease group (Fig. 7b). Correlation analysis of *AP2S1*, *STXBP1*, and *GNGT2* revealed that *AP2S1* had a significant positive correlation with central memory CD4 T cells (*r* = 0.40, *P* < 0.01), *STXBP1* had significant positive correlations with both central memory CD4 T cells (*r* = 0.42, *P* < 0.01) and central memory CD8 T cells (*r* = 0.33, *P* < 0.05), and *GNGT2* was positively correlated with myeloid-derived suppressor cells (*r* = 0.42, *P* < 0.01; Fig. 7c, Table S7). These findings suggest that these potential biomarkers could be crucial in regulating the functions and abundance of related immune cells.

### Molecular docking exploration of biomarker-drug associations in MN

**Fig. 8:**
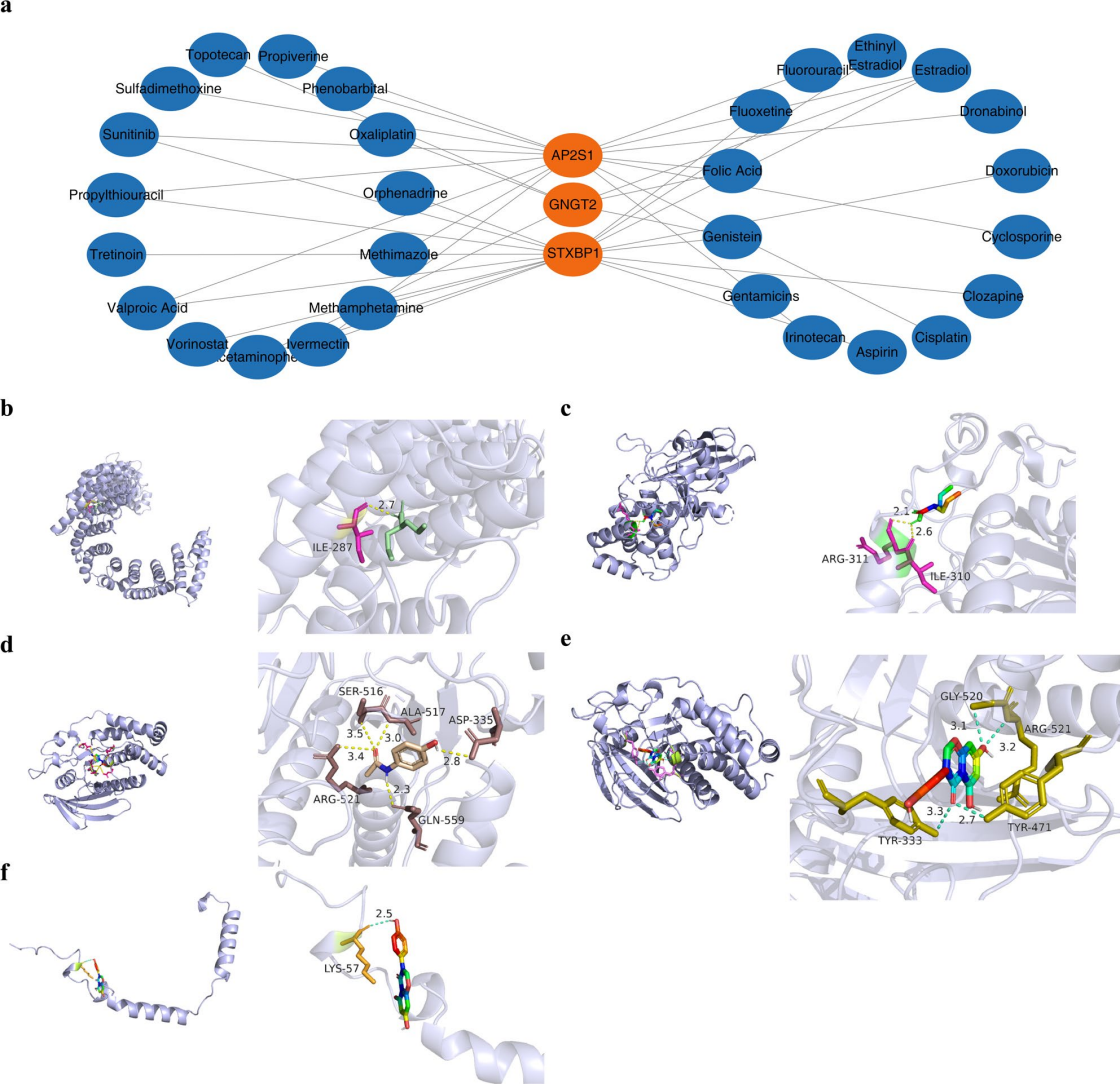
Prediction of potential therapeutic drugs and molecular docking validation. **a** Drug-gene interaction network showing potential small molecules targeting the core biomarkers. **b**–**f** 3D visualization of molecular docking results. **b** Docking of AP2S1 with Valproic Acid. **c** Docking of STXBP1 with Valproic Acid. **d** Docking of STXBP1 with Acetaminophen. **e** Docking of STXBP1 with Genistein. **f** Docking of GNGT2 with Genistein. Binding pockets are shown, and hydrogen bonds are indicated by yellow dashed lines. Binding affinity values (kcal/mol) are displayed for each complex, with negative values indicating stable binding

To prioritize candidate drugs for molecular docking validation, we focused on compounds predicted to interact with multiple potential biomarkers by the CTD database under the hypothesis that such multitarget drugs might have enhanced therapeutic potential for MN. Based on the results of CTD analysis, AP2S1, GNGT2, and STXBP1 were predicted to interact with 15, 6, and 17 drugs, respectively (Fig. 8a, Table S8). Among these, estradiol was commonly predicted for all three potential biomarkers; however, it was not selected for subsequent docking analysis because of its primary role as a hormone with complex systemic effects, as our study focused on nonhormonal agents. Instead, genistein, acetaminophen, and valproic acid were selected because they were commonly associated with at least two of the validated potential biomarkers (specifically GNGT2 and STXBP1 for genistein and STXBP1 and AP2S1 for valproic acid; Fig. 8a, Table S8). These findings suggest that the identified potential biomarkers have potential associations with specific small-molecule drugs, providing a basis for precision medicine in MN.

The study used molecular docking techniques to dock AP2S1, GNGT2, and STXBP1 with genistein, acetaminophen, and valproic acid, respectively. The results illustrated that all docking interactions were successful, and the docking scores were all ≤ − 5 kcal/mol. Specifically, the interaction between STXBP1 and genistein was the most stable, with a docking score of − 7.3 kcal/mol. The interaction between GNGT2 and genistein was also relatively stable, with a docking score of − 5.5 kcal/mol. Conversely, the interaction between AP2S1 and valproic acid was relatively weak, with a docking score of − 5.3 kcal/mol (Table 2).

**Table 2:** Molecular docking binding energies between biomarker proteins and selected small-molecule drugs

Gene	Drug	Binding energy (kcal/mol)
*AP2S1*	Valproic acid	−5.3
*STXBP*1	Valproic acid	−5.2
*STXBP1*	Acetaminophen	−6.7
*STXBP1*	Genistein	−7.3
*GNGT2*	Genistein	−5.5

In addition, the docking results of *AP2S1* and valproic acid revealed the spatial structure of their binding and marked ILE-287 as a relevant site. In the spatial structure of *STXBP1* and valproic acid, the relevant sites were ARG-311 and ILE-310. Moreover, the docking results of *STXBP1* and acetaminophen highlighted SER-516 and ASP-335 as relevant sites. The docking results of *STXBP1* and genistein revealed the importance of GLY-520 and ARG-521, and the docking results of *GNGT2* and genistein identified the relevance of LYS-57 (Fig. 8b–f). The discovery of these sites is helpful for better understanding the interaction mechanisms between related proteins and drugs, providing an important structural basis for subsequent drug design and optimization.

### RT-qPCR reveals the expression changes of *AP2S1*, *STXBP1*, and*GNGT2* in MN

**Fig. 9:**
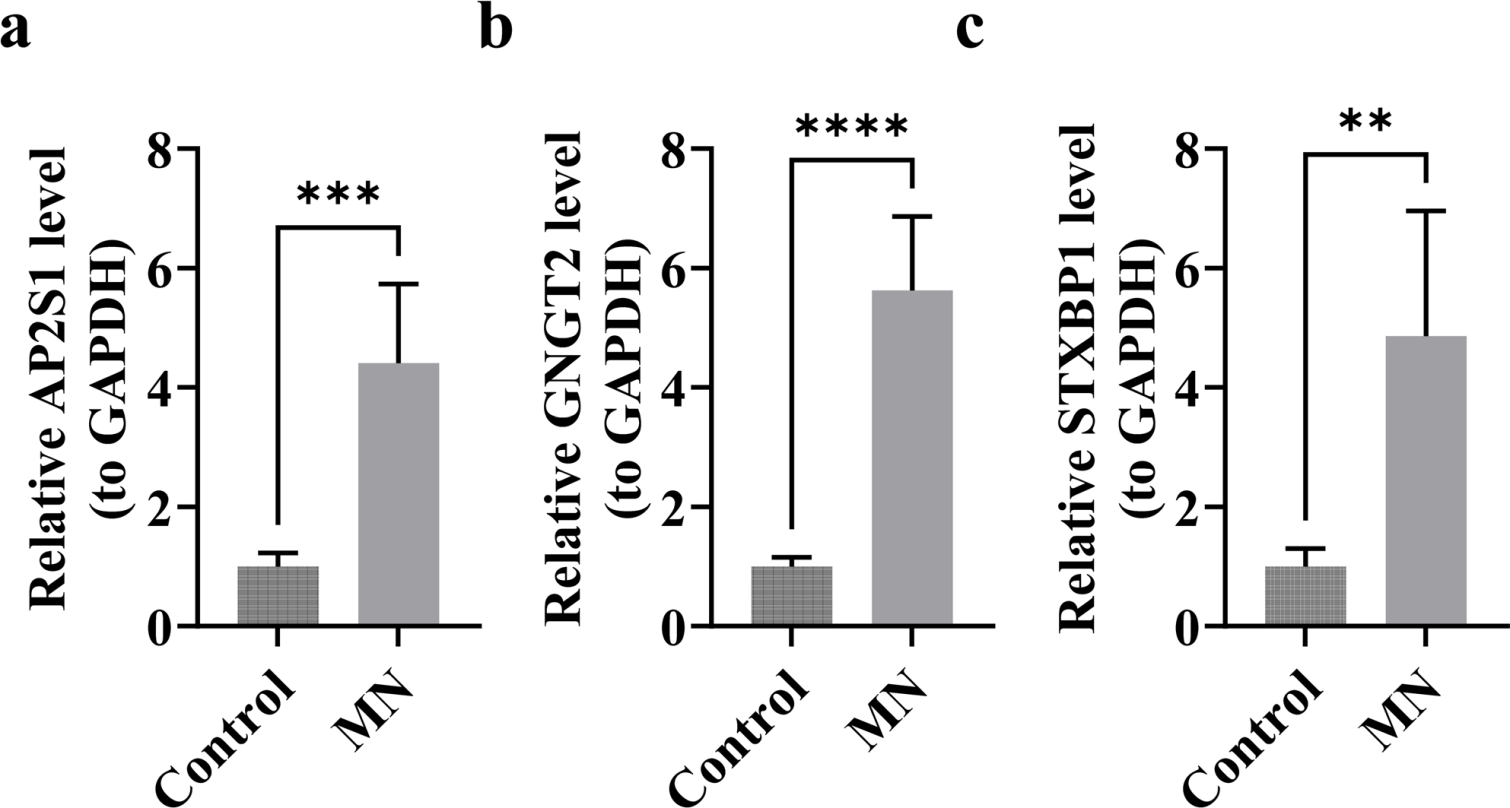
Experimental validation of core biomarker expression by RT-qPCR. **a**–**c** Relative mRNA expression levels of *AP2S1* (**a**), *STXBP1* (**b**), and *GNGT2* (**c**) in renal tissues from MN patients (*n* = 5) and healthy controls (*n* = 5). All three genes were significantly upregulated in the MN group compared to controls. Data are presented as mean ± SD. Statistical significance was determined by Student’s t-test. v > *P* < 0.01, v > *P* < 0.001, v> **P* < 0.0001

This study identified differences in the expression of *AP2S1*, *STXBP1*, and *GNGT2* between the disease and control groups using RT-qPCR. The relative expression levels of *AP2S1*, *STXBP1*, and *GNGT2* in the disease group were 4.4061 ± 1.3253, 4.8588 ± 2.1002, and 5.6223 ± 1.2428, respectively, markedly exceeding those in the control group (*P* < 0.01). These results align with those of bioinformatics analysis, suggesting that the elevated *AP2S1*, *STXBP1*, and *GNGT2* expression might play crucial roles in MN (Fig. 9a–c, Table 3).

**Table 3:** Validation of biomarker gene expression using RT-qPCR

Gene	Control	MN	*P*
*AP2S1*	1 ± 0.2263	4.4061 ± 1.3253	0.0005
*STXBP1*	1 ± 0.3037	4.8588 ± 2.1002	0.0036
*GNGT2*	1 ± 0.1575	5.6223 ± 1.2428	< 0.0001

## Discussion

In this study, we integrated transcriptomic data analysis with clinical validation to identify and characterize novel potential biomarkers for MN in the context of GABA signaling. Through a rigorous pipeline involving differential expression analysis, WGCNA, and machine learning algorithms, we pinpointed the GABA-associated genes *AP2S1*, *STXBP1*, and *GNGT2* as robust candidate biomarkers. These genes were consistently upregulated in both the training and validation datasets and were further confirmed in our clinical blood samples via RT-qPCR. Our comprehensive analyses revealed their involvement in key metabolic and signaling pathways, significant correlations with specific immune cell infiltrates, and promising binding affinities with potential therapeutic compounds. These findings provide new insights into the GABAergic signature in MN pathogenesis and offer potential targets for precision medicine.

*AP2S1*, encoding the σ subunit of adaptor protein complex 2, plays a critical role in clathrin-mediated endocytosis and intracellular vesicle trafficking [20]. Although *AP2S1* mutations are classically associated with familial hypomagnesemia with hypercalciuria and nephrocalcinosis, their roles in autoimmune glomerulonephritis remain underexplored. Our functional enrichment analysis linked *AP2S1* upregulation in MN to the CYP450 pathway, which is central to drug metabolism and oxidative stress responses [21, 22]. We hypothesize that *AP2S1* overexpression in MN might alter the endocytic turnover of membrane receptors on podocytes or immune cells, thereby dysregulating local signal transduction. Furthermore, the association of this gene with the CYP450 pathway suggests a potential mechanism by which patients with MN might exhibit altered drug metabolism, affecting the bioavailability and efficacy of therapeutic agents. This aligns with our molecular docking results highlighting strong interactions between AP2S1 and drugs such as valproic acid. Additionally, the connection of *APS21* to ribosomal function implies a role in enhancing protein synthesis demands during cellular stress or repair processes in the injured kidney [23, 24]. Future studies should investigate whether AP2S1 modulation can restore normal receptor trafficking and improve drug response in MN.

STXBP1 is traditionally recognized for its essential role in synaptic vesicle fusion and neurotransmitter release in the nervous system [20, 25, 26]. However, emerging evidence suggests that nonneuronal cells, including immune cells and renal tubular epithelial cells, utilize similar vesicular trafficking machinery for cytokine secretion and metabolic regulation. Our analysis revealed that *STXBP1* is significantly enriched in pathways related to glycine, serine, and threonine metabolism in MN. These amino acids are crucial for one-carbon metabolism, antioxidant defense (via glutathione synthesis), and cell proliferation [27, 28]. *STXBP1* upregulation might reflect an adaptive response to metabolic stress in inflamed kidneys, potentially facilitating the release of inflammatory mediators or altering mitochondrial function through oxidative phosphorylation pathways. Given its established link to neurodevelopmental disorders, the presence of STXBP1 dysregulation in MN supports the concept of a “kidney–brain axis” or shared molecular mechanisms between neuronal and renal exocytosis. Investigating whether STXBP1 inhibition can reduce inflammatory vesicle release in podocytes represents a promising avenue for future research.

GNGT2, a γ subunit of heterotrimeric G proteins, is pivotal in transducing signals from G-protein coupled receptors (GPCRs). Although previously studied mainly in retinal photoreceptors [29, 30], our findings highlighted the significant upregulation of *GNGT2* in MN and its association with cell adhesion molecules. Cell adhesion is fundamental for maintaining the integrity of the glomerular filtration barrier and regulating leukocyte migration [31, 32]. We propose that elevated GNGT2 expression might enhance GPCR signaling sensitivity in local immune cells, promoting their adhesion to endothelial cells and subsequent infiltration into the glomerulus. This mechanism could exacerbate the autoimmune response characteristic of MN. The disruption of GNGT2-mediated signaling might also interfere with renal hemodynamics and podocyte cytoskeletal organization. Thus, GNGT2 emerged as both a diagnostic marker and potential modulator of the immune microenvironment in MN, warranting further exploration of its specific downstream effectors in renal tissues.

**Fig. 10:**
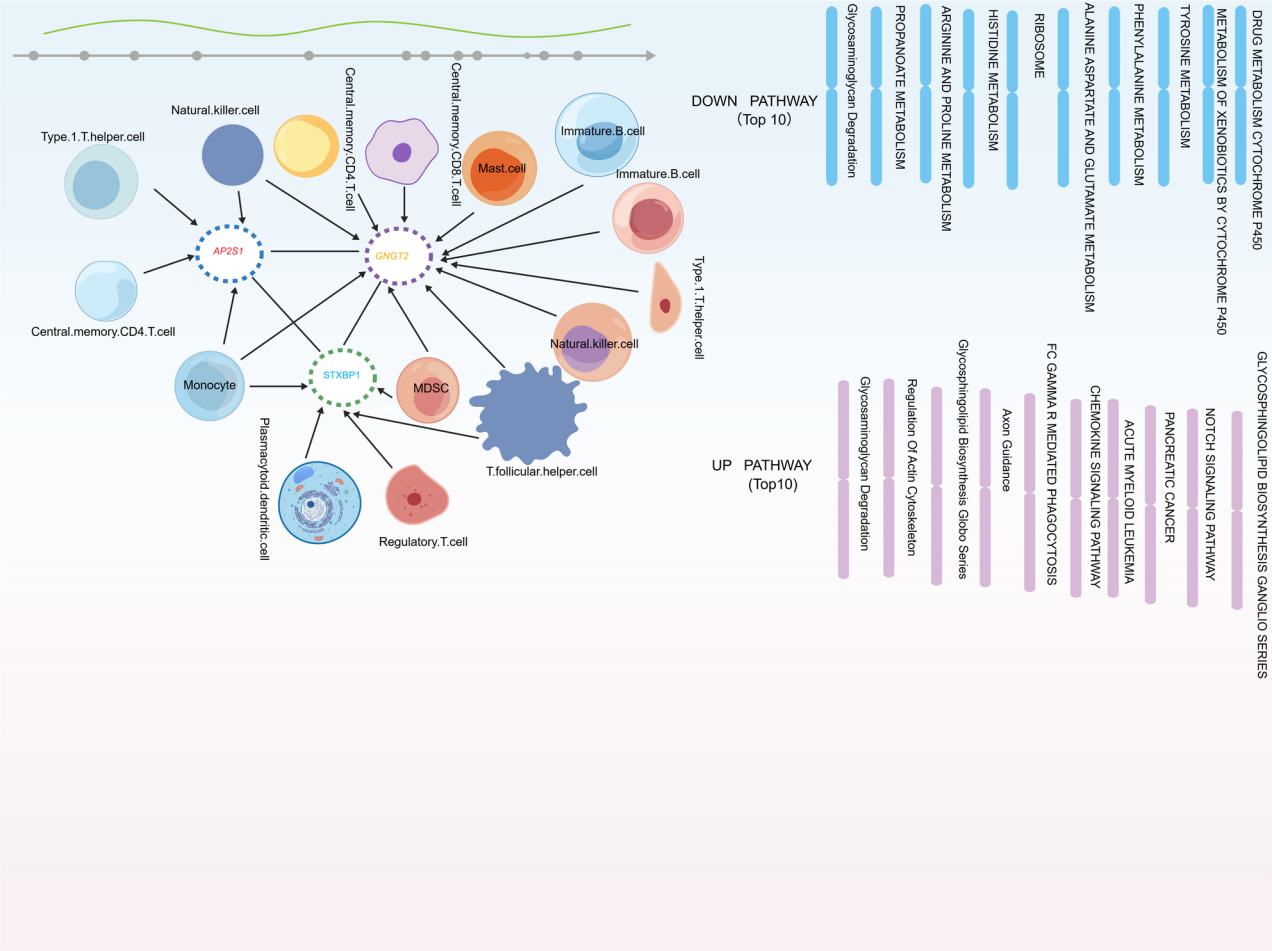
Proposed mechanistic model of *AP2S1*, *STXBP1*, and *GNGT2* in membranous nephropathy pathogenesis

Synthesizing our findings, we propose a mechanistic model (Fig. 10) in which dysregulated GABA-associated genes drive MN pathogenesis via a dual-pathway mechanism. At the cellular level, *AP2S1* and *STXBP1* overexpression disrupts podocyte vesicular trafficking, causing oxidative phosphorylation deficits and cytoskeletal instability, which lead to foot process effacement and proteinuria. Concurrently, *GNGT2* upregulation potentiates GPCR signaling and alters cell adhesion, promoting leukocyte recruitment, specifically central memory CD4 + T cells, to sustain the anti-PLA2R humoral response. Collectively, this model positions GABAergic components as pivotal hubs bridging metabolic stress, podocyte injury, and autoimmune inflammation in MN.

Our immune infiltration analysis provided further mechanistic clues, revealing significant correlations of these three candidate biomarkers with specific immune subsets, notably central memory CD4 + T cells and myeloid-derived suppressor cells. MN is fundamentally an autoimmune disorder driven by PLA2R-specific antibodies produced by plasma cells, a process heavily dependent on T cells. The positive correlations of AP2S1, STXBP1, and GNGT2 with central memory CD4 + T cells suggests that they might help sustain the long-term humoral immune response that characterizes chronic MN. Conversely, their associations with MDSCs, which typically exert immunosuppressive functions, might represent a compensatory mechanism attempting to limit tissue damage, or alternatively, a dysfunctional state that fails to control the autoimmune attack [33]. Understanding the influence of these GABAGs on the balance between effector and regulatory immune cells could uncover new strategies to restore immune tolerance in MN.

Furthermore, our molecular docking analysis identified genistein, acetaminophen, and valproic acid as potential therapeutic agents targeting these biomarkers. Genistein, a soy isoflavone, has documented anti-inflammatory and antifibrotic effects in various kidney disease models, potentially via modulation of tyrosine kinase and NF-κB pathways [34]. Valproic acid, a well-known histone deacetylase inhibitor, has exerted protective effects in models of renal fibrosis and inflammation by regulating gene expression profiles [35]. The strong binding affinities observed between these drugs and our candidate proteins suggest a plausible mechanism for their therapeutic potential. Specifically, valproic acid’s ability to modify epigenetic landscapes might directly counteract the dysregulated expression of *STXBP1* and *AP2S1*. These findings provide a theoretical basis for repurposing these existing drugs for MN treatment, although their actual efficacy and safety profiles in this specific context require rigorous preclinical and clinical validation.

The experimental validation via RT-qPCR confirmed that *AP2S1*, *STXBP1*, and *GNGT2* expression in blood samples was significantly higher in patients with MN than in controls, consistent with our bioinformatics predictions. This concordance strengthens the reliability of our multiomics approach and highlights the potential of these genes as noninvasive diagnostic markers. Unlike previous studies that focused solely on tissue biopsies, our demonstration of elevated gene expression in peripheral blood offers a more practical tool for clinical monitoring and early diagnosis.

Several important limitations of this study must be acknowledged. First, the RT-qPCR validation cohort was small (five patients and five controls), providing only preliminary proof-of-concept support rather than definitive evidence of clinical utility. More fundamentally, a biological discrepancy exists between our discovery and validation phases: the transcriptomic signatures were derived from glomerular/renal tissue datasets, whereas our experimental validation utilized peripheral blood samples. Although peripheral blood is a clinically accessible surrogate, it might not fully reflect the gene expression dynamics within the diseased renal microenvironment. Although the concordant upregulation of *AP2S1*, *STXBP1*, and *GNGT2* in both compartments suggests systemic relevance or mirrored dysregulation, this hypothesis requires validation in paired renal biopsy and blood samples from the same patients. Second, the link among GABA, these biomarkers, and MN pathogenesis remains associative rather than mechanistic. Because of data availability, we could not measure GABA or its receptors directly in renal tissues, assess functional effects in specific glomerular cells (e.g., podocytes or mesangial cells), or correlate expression with key clinical parameters such as proteinuria, serum creatinine, or anti-PLA2R titers. Third, the predicted therapeutic agents are based on computational docking, and their actual efficacy requires rigorous pharmacodynamic verification. Collectively, these constraints highlight that our work primarily generated novel hypotheses regarding GABA-associated pathways in MN. We are currently recruiting a larger cohort (target of 51 participants) to address sample size issues, and future studies must focus on functional assays in relevant renal cell types and multicenter validations with paired tissue–serum samples to establish causal roles and clinical applicability.

## Conclusion

This study identified *AP2S1*, *STXBP1*, and *GNGT2* as novel potential biomarkers for MN, supported by integrated transcriptomic analysis and preliminary RT-qPCR validation in clinical blood samples. These genes, which were consistently upregulated in patients with MN across multiple datasets, implicate GABAergic signaling in critical MN pathophysiological processes: *AP2S1* was associated with drug metabolism/CYP450 pathways, *STXBP1* was linked to amino acid metabolism, and *GNGT2* was linked to cell adhesion.

Our findings suggest three potential advances: a novel mechanistic link between GABA signaling and MN pathogenesis; the promise of a blood-based biomarker panel for noninvasive MN detection; and a theoretical foundation for precision medicine via the repurposing of compounds such as genistein and valproic acid, as indicated by molecular docking. However, the current experimental validation was performed in a small clinical cohort. Therefore, although these biomarkers and predicted therapeutics display promising translational potential, larger multicenter prospective cohorts and comprehensive functional studies (e.g., in vitro/in vivo models) are required to establish their validate. These candidates could serve as valuable starting points for future research aimed at improving MN diagnosis and developing targeted therapies.

## Supplementary Information

Supplementary Material 1. Table S1: List of Gamma-Aminobutyric Acid (GABA)-Related Genes Retrieved from the Molecular Signatures Database (MSigDB). *Data were downloaded from MSigDB v7.5.1. Only genes with official HGNC symbols were retained*. Table S2: Sequences and Specifications of Primers Used for Reverse Transcription Quantitative Polymerase Chain Reaction (RT-qPCR) Validation. *GAPDH served as the internal reference gene*. Table S3: Detailed Characteristics of the 22 Candidate Biomarkers Identified. *Intersection criteria: |log2FC| > 1, FDR < 0.05, and module membership > 0.8*. Table S4: Results of Gene Ontology (GO) and Kyoto Encyclopedia of Genes and Genomes (KEGG) Pathway Enrichment Analyses for the 22 Candidate Biomarkers. *Terms with FDR < 0.05 were considered significant*. Table S5: Enrichment Scores and Statistical Significance of Pathways Identified by Gene Set Enrichment Analysis (GSEA) and Gene Set Variation Analysis (GSVA). Table S6: Predicted MicroRNA (miRNA)-mRNA Interactions and Competing Endogenous RNA (ceRNA) Regulatory Networks Associated with Membranous *Only pairs with negative correlation (P < 0.05) in MN datasets are shown*. Table S7: Differential Analysis of Immune Cell Infiltration Scores Between Membranous Nephropathy Patients and Healthy Controls. Table S8: List of Potential Therapeutic Small Molecules Targeting the Identified Candidate Biomarkers Predicted by Molecular Docking and Drug-Gene Interaction Databases.

## Data Availability

The data presented in this study are available in the GEO database at [https://www.ncbi.nlm.nih.gov/geo/], reference numbers GSE200828 and GSE115857.

## Abbreviations

MN: Membranous Nephropathy
GABA: Gamma-Aminobutyric Acid
WGCNA: Weighted Gene Co-expression Network Analysis
AUC: Area under the curve
PPI: Protein-Protein Interaction
MMP: Matrix metalloproteinase
GSEA: Gene Set Enrichment Analysis
GSVA: Gene Set Variation Analysis
GLM: Generalized linear model
RT-qPCR: Reverse Transcription Quantitative Polymerase Chain Reaction
PMN: Primary MN
SLE: Systemic Lupus Erythematosus
MSigDB: Molecular Signature Database
DEGs: Differential Expression Gene
WGCNA-MNKGs: WGCNA-MN Key Genes
IVW: Inverse variance weighted
GO: Gene Ontology
BP: Biological Process
MF: Molecular Function
CC: Cellular Component
KEGG: Kyoto Encyclopedia of Genes and Genomes
LASSO: Least Absolute Shrinkage And Selection Operator
miRNAs: MicroRNAs
lncRNAs: Long Non-Coding RNAs
CTD: Comparative Toxicogenomics Database
MDSC: Myeloid-Derived Suppressor Cells
